# High-Temperature unfolding of a trp-Cage mini-protein: a molecular dynamics simulation study

**DOI:** 10.1186/1742-4682-2-7

**Published:** 2005-03-11

**Authors:** Aswin Sai Narain Seshasayee

**Affiliations:** 1 Centre for Biotechnology, Anna University, Chennai 600025, India

## Abstract

**Background:**

Trp cage is a recently-constructed fast-folding miniprotein. It consists of a short helix, a 3,10 helix and a C-terminal poly-proline that packs against a Trp in the alpha helix. It is known to fold within 4 ns.

**Results:**

High-temperature unfolding molecular dynamics simulations of the Trp cage miniprotein have been carried out in explicit water using the OPLS-AA force-field incorporated in the program GROMACS. The radius of gyration (Rg) and Root Mean Square Deviation (RMSD) have been used as order parameters to follow the unfolding process. Distributions of Rg were used to identify ensembles.

**Conclusion:**

Three ensembles could be identified. While the native-state ensemble shows an Rg distribution that is slightly skewed, the second ensemble, which is presumably the Transition State Ensemble (TSE), shows an excellent fit. The denatured ensemble shows large fluctuations, but a Gaussian curve could be fitted. This means that the unfolding process is two-state. Representative structures from each of these ensembles are presented here.

## Background

Understanding the mechanisms behind protein folding, which is one of the most fundamental biochemical processes, is proving to be a challenging task for biochemists and biophysicists. Recent developments in instrumentation and methodology have enabled us to take major steps forward in comprehending the dynamics of proteins and peptides at the molecular level. Protein engineering methods such as Phi-value analysis [1] and various spectroscopic techniques such as NMR have made the task more practicable.

Proteins are composed of two major secondary structural elements, helices and sheets, which, along with loops, pack together to form super-secondary and tertiary structures. Trp cage is a novel, and a highly stable, mini-protein fold. A 20-residue Trp-cage miniprotein has been designed [2]. It has the sequence NLYIQWLKDGGPSSGRPPPS. While residues 1–9 form an alpha helix, residues 10–15 form a 3,10 helix. W6 is caged by the C-terminal poly-proline stretch. D9 and R16 are involved in a stabilizing salt-bridge interaction.

Molecular dynamics simulations, which make use of classical Newton mechanics to generate trajectories, are playing an ever-expanding role in biochemistry and biophysics due to substantial increases in computational power and concomitant improvements in force fields. In particular, the contribution of such studies to protein folding is immense [1]. As pointed out by Fersht and Dagget, molecular dynamics simulations are capable of unraveling whole protein folding / unfolding pathways [1]. Indeed, simulation techniques have been widely used for studying helices and sheets. Today, folding simulations of more-than-model peptides are being carried out on high-power computers.

Despite being a new mini-protein construct, the Trp cage motif has attracted considerable computational analysis. Folding simulations of this protein in explicit water have been carried out using what is known as the Replica Exchange Method. A two-state folding mechanism has been proposed and free energy surfaces have been determined [3]. Moreover, a few folding simulations of have been carried out using implicit solvation models [4-6]. In this article, the results of a high-temperature unfolding simulation of the Trp-cage mini-construct are presented. Three separate structural clusters are identified: the close-to-native-state cluster, the intermediate cluster and the denatured ensemble. These clusters, considered in terms of their radii of gyration, are shown to be Gaussian ensembles. Structural features representing each of these ensembles are also illustrated.

## Results and Discussion

Molecular dynamics simulations of the Trp-cage mini-protein construct (PDB ID: 1L2Y) were carried out using the OPLS-AA force-field incorporated in the freely available program, GROMACS. The simulations were carried out at 498 K, at which temperature the unfolding process is favored. This temperature provides a good description of the unfolding process, at least in respect of CI2 and the homeodomain of engrailed [7]. It is also much higher than the melting temperature determined by experiment (315 K) or through replica-exchange simulations (400 K) [3].

**Figure 1:**
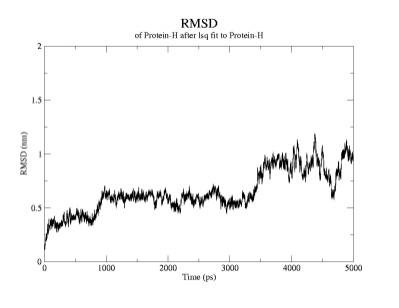
Time evolution of the root mean square deviation (nm) with reference to the starting structure.

**Figure 2:**
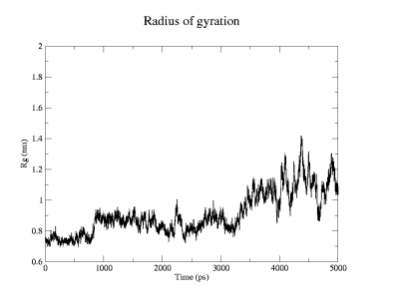
Time evolution of the radius of gyration (nm)

**Figure 3:**
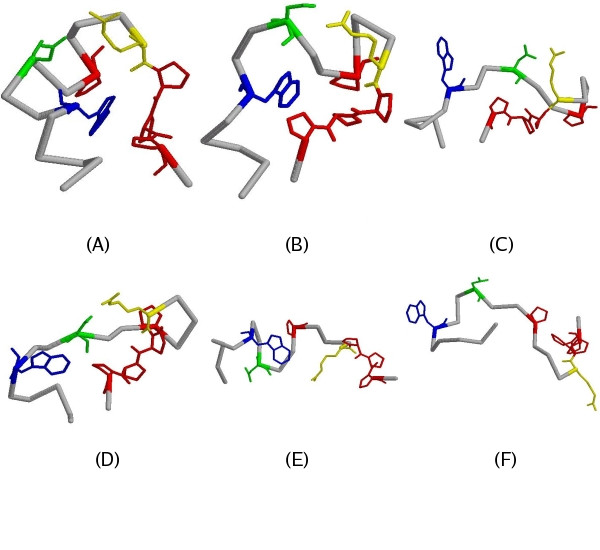
Representative structures from the folding pathway obtained after (A) 0 ps (B) 700 ps (C) 1000 ps (D) 2500 ps (E) 4000 ps (F) 5000 ps. Structures A and B belong to the first ensemble; C and D to the second and E and F to the third. Color code: Pro: Red; Trp: Blue; Asp: Green; Arg: Yellow

It can be seen that the RMSD (figure 1) of the evolving structure with reference to the starting structure increases rapidly in the first 40 ps, during which time the only structural change observed is denaturation of the 3,10 helix. This is followed by rapid unwinding of the second and third turns of the helix. While the third turn unwinds within 200 ps, the second turn remains intact for a little longer and remains visible until 250 ps. The first helical turn remains stable until about 800 ps after which it also denatures. During this time period W6 begins to move out of the cage that is formed by the prolines. The above listed processes are not adequately reflected by the time-evolution of the Rg (figure 2) and are all categorized as close-to-native-state ensemble. Representatives from this ensemble are shown in figure 3a and 3b.

After 800 ps, there is a jump in the values of both RMSD and Rg. The new value remains constant until about 3200 ps. This state is characterized by complete annihilation of the cage. The W6 is released from the Pro cage and becomes completely "solvent-exposed". It must be noted that the use of the term "solvent exposed" is not entirely appropriate in this context as there is no real change in the solvent-accessible surface area of the W side-chain. However, the point is that, this W is no longer protected by the proline cage. Native contacts are retained in the form of a salt-bridge between D9 and R16. Representatives of this ensemble are shown in figure 3c, d. In fact, the folding simulations carried out by Ruhong Zhou [3] point to an intermediate state characterized by the single salt-bridge interaction. This state, which is the only intermediate state observable, may be the transition state ensemble (TSE). This would mean that the unfolding process is two-stage and is the reversal of the folding process. In order to assess whether this state is indeed the TSE, lower temperature simulations at 293 K were performed. Eight structures were randomly obtained from this ensemble and the simulations were carried out for 5 ns on each of these structures. The progress of each simulation was monitored using Rg. The idea is that, at temperatures favoring the folding process, structures from the TSE roll down towards the native state with a probability of approximately 0.5, assuming a two-state process [1]. Of the eight simulations, three simulations showed a drastic fall in the Rg, indicating a collapse towards the native state. In a fourth simulation, there was a slight decrease in the Rg, which was not drastic, but still implying a fall towards the native state. In the other four simulations, a significant jump in the Rg was observed, indicating a tendency towards the unfolded conformation. These observations show that this ensemble is, most probably, the TSE.

After 3200 ps, a further jump in RMSD and Rg is observed leading to a state where these values fluctuate markedly. This highly disordered state, showing a measure of heterogeneity, is the denatured ensemble, in which the salt-bridge interaction that characterized the intermediate state is also lost. There is a significant jump in the distance between the Asp9 and Arg 16 sidechains after this time. As a result, there are no native contacts in this state. This is represented by structures in figures 3e and 3f.

**Figure 4:**
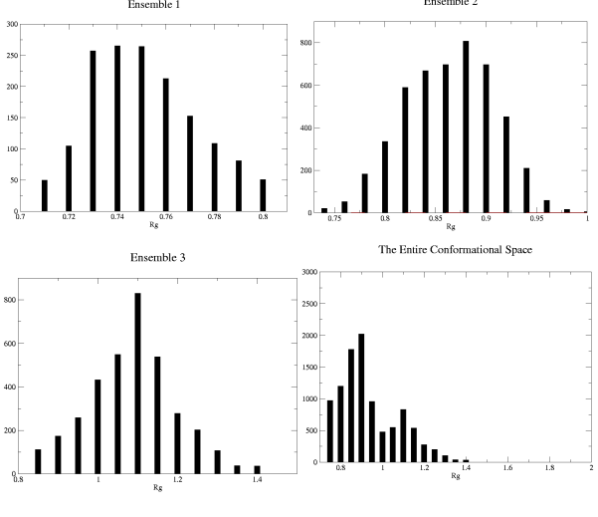
Distributions of Radius of gyration for (A) Ensemble 1 (B) Ensemble 2 (C) Ensemble 3 (D) Entire range of structures.

In this manuscript, I also discuss a new method for identifying sufficiently populated states during the course of an MD simulation. The idea is that each state is to a large extent topologically different from any other state and can be characterized by an approximately Gaussian distribution of the radius of gyration. This is to be expected because each state lies at a defined height in the free-energy well. In this simulation it can be observed that transitions from one state to another are characterized by a significant jump in the radius of gyration. The distribution of the radius of gyration was determined for each of the three states and for the entire time-evolving system. For each of the three ensembles and for the entire time duration, the distribution was calculated over the ranges of values shown in table 1. It was found that Gaussian-like curves could be fitted for the three ensembles taken separately, while the distribution for the entire system was highly skewed (figure 4). The slight skew in the curve for the close-to-native state ensemble might be due to the inability to sufficiently demarcate the helix unwinding stages in the plot.

**Table 1:** Rg range and time corresponding to each state seen in the simulation

***Ensemble***	***Time (ps)***	***Rg range (nm)***
Native	0–800	0.7 – 0.8
TSE	800–3200	0.72 – 1
Unfolded	3200–5000	0.8 – 1.4
Entire range	0–5000	0.7 – 1.4

## Conclusion

High-temperature unfolding molecular dynamics simulations of a Trp cage miniprotein construct have been carried out. This has shown that the process is two-stage, akin to the folding process results [3]. The three ensembles, including the TSE, are shown to be Gaussian with respect to their Rg values.

## Methods

The starting structures for the simulations were obtained from PDB 1L2Y [3]. The first three models were used to carry out the 5 ns simulations and similar results were obtained with each. Results presented here correspond to model 1. All simulations were carried out using GROMACS 3.2 [8,9], running on a single Fedora Linux system. The OPLS-AA force field was used. The peptide was solvated in a box containing approx. 500 water molecules [10]. Periodic boundary conditions were employed to eliminate surface effects. Energy minimization with a tolerance of 2000 kJ/mol/nm was carried out using the Steepest Descent method. All bonds were constrained using LINCS [11]. The system was loosely coupled to a temperature bath (at 498 K or 293 K) using Berendsen's method [12]. Berendsen's pressure coupling was used. Long-range electrostatics was handled using the PME method [13]. All potential cut-offs were set at 1 nm. The final MD simulations were carried out with a time-step of 2 fs and without any position restraints. All analyses were conducted using programs built within GROMACS. The RMSD values were obtained from a least square fit of the respective non-hydrogen atoms (main-chain and side-chain). The radius of gyration was also calculated for the whole protein minus hydrogens as an indicator of the compactness of the overall structure. The compiled DSSP [14], which was downloaded separately and run from GROMACS, was used to calculate secondary structure formation.

## Competing Interests

The author(s) declare that they have no competing interests.
